# Sheep as a Potential Model of Intradiscal Infection by the Bacterium *Cutibacterium acnes*

**DOI:** 10.3390/vetsci8030048

**Published:** 2021-03-16

**Authors:** Erin C. Coscia, Nader S. Abutaleb, Bradley Hostetter, Mohamed N. Seleem, Gert J. Breur, Robyn R. McCain, Christa J. Crain, Ondrej Slaby, Manu N. Capoor, Andrew McDowell, Fahad S. Ahmed, Viju Vijayanpillai, Sanjeev K. Narayanan, Michael F. Coscia

**Affiliations:** 1 College of Osteopathic Medicine, Marian University, Indianapolis, IN 46222, USA; ecoscia881@marian.edu; 2 Department of Comparative Pathobiology, College of Veterinary Medicine, Purdue University, West Lafayette, IN 47907, USA; nabutale@purdue.edu (N.S.A.); mseleem@purdue.edu (M.N.S.); gert.j.breur@gmail.com (G.J.B.); vijayanp@purdue.edu (V.V.); sanjeev@purdue.edu (S.K.N.); 3 Center for Diagnostic Imaging, Carmel, IN 46032, USA; bhostetter@cdirad.com; 4 Department of Biomedical Sciences and Pathobiology, Virginia-Maryland College of Veterinary Medicine, Virginia Polytechnic Institute and State University, Blacksburg, VA 24061, USA; 5 Center for Comparative Translational Research, Purdue University, 625 Harrison Street, West Lafayette, IN 47907, USA; rrmccain@purdue.edu (R.R.M.); ccrain@purdue.edu (C.J.C.); 6 Central European Institute of Technology (CEITEC), Masaryk University, 625 00 Brno, Czech Republic; ondrej.slaby@ceitec.muni.cz (O.S.); fsa526@gmail.com (F.S.A.); 7 Laboratory of Bacterial Pathogenesis and Immunology, Rockefeller University, 1230 York Avenue, New York, NY 10065, USA; mcapoor@rockefeller.edu; 8 Nutrition Innovation Centre for Food and Health (NICHE), School of Biomedical Sciences, Ulster University, Coleraine BT52 1SA, UK; a.mcdowell@ulster.ac.uk; 9 OrthoIndy, 8450 Northwest Blvd, Indianapolis, IN 46278, USA

**Keywords:** *Cutibacterium acnes*, sheep model, spinal intervertebral discs, percutaneous injections, bacterial discitis

## Abstract

The anaerobic bacterium *Cutibacterium acnes* has been increasingly linked to the development of degenerative disc disease (DDD), although causality is yet to be conclusively proven. To better study how this organism could contribute to the aetiology of DDD, improved animal models that are more reflective of human disc anatomy, biology and mechanical properties are required. Against this background, our proof-of concept study aimed to be the first demonstration that *C. acnes* could be safely administered percutaneously into sheep intervertebral discs (IVDs) for in vivo study. Following our protocol, two sheep were successfully injected with a strain of *C. acnes* (8.3 × 10^6^ CFU/disc) previously recovered from a human degenerative disc. No adverse reactions were noted, and at one-month post inoculation all triplicate infected discs in our first animal grew *C. acnes*, albeit at a reduced load (5.12 × 10^4^ to 6.67 × 10^4^ CFU/disc). At six months, no growth was detected in discs from our second animal indicating bacterial clearance. This pilot study has demonstrated the feasibility of safe percutaneous injection of *C. acnes* into sheep IVDs under fluoroscopic guidance. The design of follow-up sheep studies to investigate the potential of *C. acnes* to drive pathological changes within infected discs should now be pursued.

## 1. Introduction

According to the Global Burden of Disease study of 2017, low back pain (LBP) is the world’s leading cause of disability [1]. It is estimated that 80% of Americans will experience LBP during their lifetime, and this incidence is predicted to increase due to ageing populations and current demographics such as sedentary lifestyles and obesity [2]. Although the etiology of low back pain is multifaceted, there is a strong correlation with degeneration of the intervertebral disc (IVD) [3]. Curative treatments for degenerative disc disease (DDD) are not available, as the complex pathologic mechanisms, including disc nutritional factors, cytokines, biomechanical factors, and genetic factors are not fully understood.

Over the last 20 years, evidence has accumulated to suggest that infection of IVDs especially with the anaerobic bacterium *Cutibacterium acnes* (previously *Propionibacterium acnes*) may have an important role to play in the development of DDD and discogenic pain [4,5,6,7,8,9,10,11,12,13,14]. This organism, which is a substantial component of the normal microbiota on human skin, is a low-grade opportunistic pathogen most famously described for its association with the inflammatory skin condition acne vulgaris [15]. It is, however, also a cause of osteomyelitis, eye, dental and indwelling medical device-related infections, and has been linked to prostate disease and sarcoidosis [16,17,18,19,20,21,22]. *C. acnes* has been observed in IVDs with a biofilm-like morphology that precludes it presence in such samples due to surgical contamination [23,24]. It has also been reported that human IVDs associated with *C. acnes* are more likely to be characterised by type I modic changes (MC) in the endplates on magnetic resonance imaging (MRI) scans [25,26,27]. Animal studies have also shown that *C. acnes* infection of rabbit discs induces degeneration and inflammation of the endplate region, presenting as MCs [28,29]. Despite these observations, the role that *C. acnes* may play in DDD remains controversial, and continued studies are required, especially using appropriate animal models, to help establish causality. More generally, the development of accurate animal models of DDD would also provide more meaningful insight into the pathophysiology of DDD, as well as a vehicle for testing potential therapeutics [30].

Commonly used animal models for disc research include rabbits, dogs, and sheep, as well as the caudal discs of rats and cows. The majority of these models (rats, pigs, rabbits, and cows) are, however, flawed in their application to DDD in humans as they differ from human lumbar discs in terms of cell composition, size, and mechanical loading [31]. For example, many species (rats, pigs, cats, rabbits, and some dogs) retain notochordal cells into adulthood, and these appear to help maintain the IVD [32]. In contrast, humans and chondrodysplastic canines lose notochordal cells postnatally, and both develop spontaneous DDD [32].

Sheep discs closely approximate to human discs in size, experience similar biomechanical stresses, and lose notochord cells in early adulthood [30,31,32,33,34]. Furthermore, sheep recover well from surgical interventions and are relatively easy to maintain long term [30]. For these reasons, sheep have frequently served as models for DDD as well as device implantation and cellular therapy investigations [30]. Additionally, the use of intermediate or large sized animals helps to decrease errors associated with “scaling up” of medication doses and therapies for use in subsequent human clinical studies [34].

As a sheep model of *C. acnes* infection of IVDs could be extremely valuable to study the role of this bacterium in DDD, the aim of this proof-of-concept investigation was to be the first to demonstrate that *C. acnes* could be safely administered percutaneously into sheep IVDs to establish a short term or chronic infection.

## 2. Materials and Methods 

### 2.1. Animals

Mature Dorset cross ewes (approximately 60 kg; 2.5 years of age) were included in this study, each bearing a permanent United States Department of Agriculture (USDA) tag to prevent any animal misidentification (#USDA 1283 and 1286). Sheep were individually housed in 5 × 5 foot pens, and fed grass hay twice per day; no other foods or supplements were utilised. This study was approved by Purdue University’s Institutional Animal Care and Use Committee [IACUC] and all experiments were performed in accordance with relevant guidelines and regulations.

### 2.2. Anesthesia

A standard anesthesia protocol was used for percutaneous intradiscal injections and MRI. Sheep were premedicated with xylazine (AnaSed; Akorn, Lake Forest, IL, USA; 0.03 mg/kg IV) and butorphanol (Torbugesic; Zoetis, Kalamazoo, MI USA, 0.01 mg/kg IV) and induced with a combination of ketamine (VetaKet; Akorn, 2.2 mg/kg IV) and diazepam (Hospira, Lake Forest, IL USA, 0.06 mg/kg IV). After local application of a few drops of a 2% lidocaine (AnaSed; Akorn) solution on the vocal folds, sheep were intubated and anesthesia was maintained using isoflurane. Throughout anesthesia, lactated Ringer’s solution was administered (10 mL/kg/hr). Post-operatively, buprenorphine (Par Pharmaceutical, Chestnut Ridge, NY USA, 0.005 mg/k IV) and flunixin meglumine (Merck & Co., Kenilworth, NJ, USA, 1.1 mg/kg IM) were administered for analgesia.

### 2.3. MRI

All scans were performed using a 1.5 Tesla GE Signa LX (General Electric, Milwaukee, WI, USA) magnet with the sheep in dorsal (supine) recumbancy; at each session, all scans were reviewed by three physician board certified radiologists. Under general anesthesia, a baseline MRI scan of their spine was performed, and during the same anesthetic period, selected lumbar discs of each sheep were percutaneously injected by a board certified, physician neuroradiologist (BH). Once a month, again under general anesthesia, an MRI scan of the lumbar spine was repeated. MRIs were not performed at three and five months for sheep #1286 due to very cold weather, which prohibited the transfer of the sheep.

### 2.4. Bacterial Injectate

The *C. acnes* strain selected for this study belonged to the type IA_1_ phylogroup (*C. acnes* subsp. *acnes*) (PD271) and was previously cultured from human pathologic disc material; this was provided by the Central European Institute of Technology (Brno, Czech Republic (OS)). The bacterium was cultured anaerobically in brain heart infusion broth at 37 °C until logarithmic growth phase. The bacterial cells were then washed and resuspended in phosphate buffered saline (PBS) (Fisher Scientific, Waltham, MA, USA). Bacterial counts were carried out on modified Clostridial Reinforced Agar (CRA) (Becton, Dickinson and Company, Cockeysville, MD, USA). Each IVD was injected with inoculum (~0.1 mL) containing ~ 8 × 10^7^ colony forming units (CFU)/mL which was similar to that utilised in previously published rabbit animal model studies with this bacterium [28,29].

### 2.5. Injection Protocol

The intra-discal injection protocol described below, which was previously developed on rabbits [28,29,35], was practiced on a sheep from an unrelated study. Instead of bacterial injectate, a radiopaque contrast medium (Omnipaque 180 Iohexol, GE Healthcare/McKesson Medical-Surgical, Marlborough, MA, USA) was utilised, then the location of the injections was verified using computerised tomography (GE VCT 64 slice; General Electric), to ensure that the discograms were anatomic, as desired (Figure S1).

Sheep were anesthetised and their lumbar areas clipped and scrubbed with chlorhexidine using standard techniques. After the sheep was placed in perfectly symmetric ventral (prone) recumbancy, sterile surgical drapes were applied. Then, needles were percutaneously inserted in selected discs under fluoroscopic guidance. Using antero-posterior and lateral “C”-arm (GE OEC 9900 Elite, GE OEC Medical Systems, Inc., Salt Lake City, UT, USA) fluoroscopic radiographic verification, five separate six inch 25/20 gauge coaxial Quincke spinal needles (#183109; Halyard Health, Apharetta, GA, USA) were inserted from a right posterolateral approach. The 20-gauge hollow introducer needle was started approximately 5.5 cm lateral to the mid-line and angled medially (toward the spine) at an approximate 30° angle to the vertical, exactly in line with the disc itself, and stopped at the outer edge of the disc annulus. A six inch, 25-gauge Quincke hollow needle (also Halyard Health) was then passed through the introducer needle and into the centre of the nucleus pulposus region of the disc. A 1 mL Hamilton Gastight syringe (#81301; Hamilton Company, Reno, NV, USA) was used to hold the inoculum, which was kept under anaerobic conditions until the injection step. Discs L2-3, L4-5, and L6-7 were injected with the *C. acnes* inoculum in all animals, whereas L3-4 had a needle insertion only (no injectate) and L5-6 was injected with saline (0.12 mL).

### 2.6. Euthanasia and Post Mortem Procedures

Sheep were euthanised with sodium pentobarbital (Euthanasia Solution; MWI, Boise, ID, USA, or Euthasol; Virbac AH, Inc., Fort Worth, TX, USA) 390 mg/mL IV (15 mL) according to the 2020 AVMA Guidelines for the Euthanasia of Animals [36]. One sheep (USDA#1283) was euthanised 1 month after injection to investigate if infection was still present in the relative short term, while the other was sacrificed (USAD#1286) at 6 months to determine if a long term or chronic infection had established. Immediately after euthanasia the lumbar spine was isolated, and the surrounding muscles and tendons were removed. Then each individual disc was aseptically retrieved in its totality.

### 2.7. Disc Tissue Sampling

Harvested discs were extensively homogenised in sterile PBS using an Omni Tissue Homogenizer (Omni International, Keenesaw, GA, USA) under anaerobic conditions for both anaerobic and aerobic bacterial culture. To determine the bacterial load in the discs, the homogenate was serially diluted in sterile PBS and aliquots (4 L) of each dilution plated on modified CRA plates for incubation anaerobically at 37 °C for an extended period of time before bacterial enumeration. For our sheep USAD #1286 at six months, disc tissue was also evaluated by Gram staining.

### 2.8. Statistical Analysis

Statistical analysis was performed using the Wilcoxon-matched-pairs signed rank test (2-tailed).

## 3. Results

### 3.1. Percutaneous Injection of C. acnes into Sheep IVDs under Fluoroscopic Guidance

**Figure 1:**
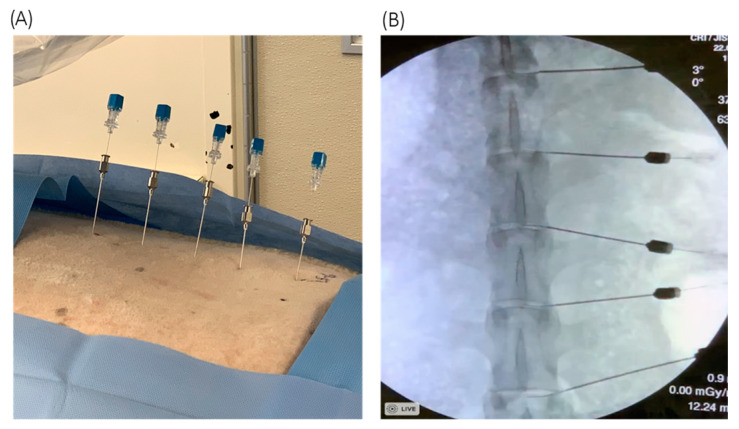
(**A**) 25-gauge needles within the 20-gauge introducer needles, serially spaced into the lumbar discs. (**B**) Fluoroscopic radiograph verifying needle placement in the AP plane.

Under fluoroscopic guidance, six inch 25/20-gauge hollow coaxial spinal needles were able to accurately penetrate percutaneously into the nucleus pulposus of sheep discs prior to the introduction of a *C. acnes* inoculum via a Hamilton syringe (Figure 1). The percutaneous injection technique, in the hands of a highly experienced neuroradiologist, was straightforward and easily documented with biplane C-arm fluoroscopy (Figure 1B).

### 3.2. Animal Welfare

No adverse reactions were observed with either animal upon intradiscal injection, and the general anesthesia and MRIs were uneventful with no complications. There was no sign of constricted mobility after injection and the sheep were independently mobile and eating shortly after the procedure. In the first sheep used for a one-month infection (USDA#1283), a single disc (L5-6) did generate a “giving way” sensation when inoculated with sterile saline, possibly due to internal acute annular fissure formation.

### 3.3. Evidence of Short-Term C. acnes Colonisation

**Figure 2:**
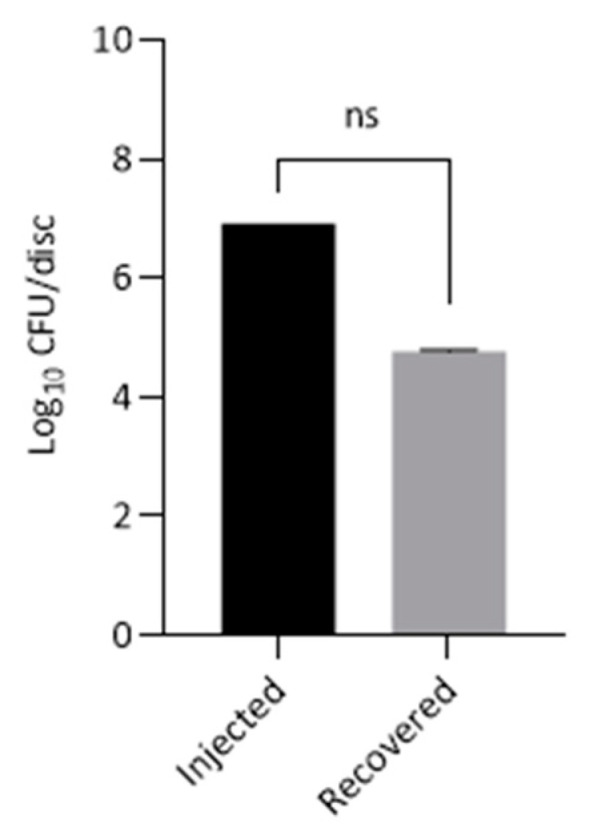
Growth of *C. acnes* from 3 × discs of the same sheep (USDA#1283) four weeks after initial inoculation. Data is presented as mean ± SEM of log_10_ CFU per disc. Statistical analysis was performed for paired samples.

The sheep sacrificed at one-month post inoculation (USDA#1283) demonstrated the presence of *C. acnes* in all three IVD tissues after CRA plate culture. Although the total numbers of *C. acnes* cultured (5.12 × 10^4^–6.67 × 10^4^ CFU per disc) were less than the original 8 × 10^6^ CFU per disc inoculums, this was not statistically significant (Figure 2).

With our second animal sacrificed at six-months post inoculation (USDA#1286), no bacteria were detected in any of the infected IVD tissues by anaerobic and aerobic culture or Gram staining. For both animals, control discs injected with sterile saline or needle insertion only were all culture-negative.

### 3.4. MRI 

**Figure 3:**
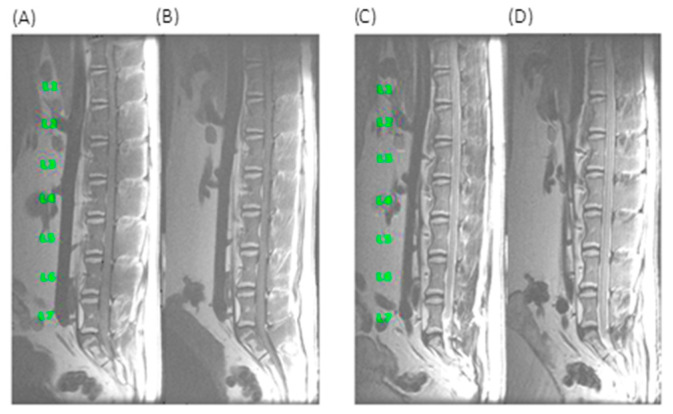
One month MRI scans for sheep USDA#1283. (**A**) T1 weighted baseline (**B**) One month T1 weighted (**C**) T2 weighted FS baseline (**D**) one month T2 weighted FS.

The one month MRI scan for the animal sacrificed at this endpoint remained unchanged from the pre-injection baseline (Figure 3).

Similarly, for the second sheep sacrificed at 6 months (USDA#1286) no MRI changes were observed from baseline at 1,2,4 and 6-month endpoints within the disc space and/or adjacent vertebral bodies (data not shown).

## 4. Discussion

The objective of this proof-of-concept study was to demonstrate that percutaneous inoculation of sheep lumbar IVDs with *C. acnes* can be performed safely and is a feasible approach for future investigations. In this regard, our work was successful and the infected sheep suffered no adverse reactions during the procedures. Due to the pilot nature of this study, and the use of large animals that were required to be sacrificed at each endpoint for disc analyses, we made the decision that we would only utilize two sheep in this phase of our investigations which we note is a limitation of the work. This was based on ethical grounds and to minimize the stress and cost to the animals, and to keep to the principle of the accepted 3Rs (Replacement, Reduction, Refinement). While this meant that only one animal was used for each time point, we balanced this with the infection of three discs per animal for triplicate observations. While the latter could be considered ‘technical replicates’ rather than true biological replicates, we feel this approach was the right balance given we had no prior knowledge on how the sheep would react to bacterial infection or indeed the viability of the experiments.

Our ability to perform percutaneous injections into the nucleus pulposus of the sheep lumbar discs is consistent with the findings of Elliott and colleagues in their very thorough study of needle diameter effects in animal models of disc degeneration [37]. They found that if a needle’s diameter to disc height was less than 40% there should be minimal annulus damage, although variable but nonsignificant minor effects were observed with needle:height ratios between 25–40%. Due to the stiffness required in our study, we felt that a 25-gauge needle would be necessary for our experiments to transition through the skin, fascia, and musculature to reach the surface of the annulus and continued entry into the nucleus pulposus. While a 27-gauge needle was used with no appreciable damage in sheep by Elliott et al. [37], a 25-gauge needle was not specifically evaluated. However, as 25-gauge = 0.455 mm diameter, and the average disc height of the sheep used in their study was 3.93 mm, this would only equate to 0.455/3.93 = 12%. On this basis, we believed a 25-gauge needle insertion would not cause any appreciable harm to our discs and our MRI scans of puncture alone seem to validate that this was correct. Moving forward, we recommend the use of 25-gauge needles since they are malleable and strong enough to penetrate the ovine AF whilst causing minimal damage.

Beyond the demonstration that successful inoculation of sheep IVDs with *C. acnes* was possible without significant tissue disruption, our results also revealed that once injected, *C. acnes* can survive within the disc for at least one month, although the bacterial load measured after this period did reduce. At the six-month endpoint, however, no bacteria were evident based on culture or Gram staining of tissue. The apparent absence of *C. acnes* after this period likely reflects its clearance from the IVD tissue. While inadequate tissue processing to vigorously disrupt any potential biofilm or bacterial cells existing intracellularly within disc tissue may also lead to reduced or false-negative culture counts, this was not the case in our study as all tissue was extensively homogenised prior to agar plate culture. The absence of bacterial growth at six months may also reflect our use of only a single animal for this endpoint. It is very possible that with other sheep displaying a different biological response to infection, or indeed a different *C. acnes* strain, the results may well be different. We note, however, that in previous studies where *C. acnes* has been used to investigate disc changes in rabbits and rats over prolonged time intervals, bacterial loads after initial inoculation were not studied [28,29,38]. As a consequence, it is unclear the extent to which, if any, the bacterium was still present in these animals at completion of the experiments. We can speculate that in humans, DDD due to *C. acnes* may occur based on cycles of infection, clearance and reinfection in compromised discs of susceptible individuals, or an initial infection may stimulate a chronic inflammatory response that persists after the organism has disappeared. Interestingly, a previously described mouse model of prostate infection with *C. acnes* found that while the bacteria could still be detected in the dorsal prostate at weeks one and two post-inoculation, at week eight no organisms were present based on immunohistochemistry analysis despite the persistence of chronic inflammation in most animals [39]. Also, in a rat model of prostate infection with *C. acnes*, bacterial counts were similarly found to fall over time to very low levels at six months [40]. On the basis of these previous small animal data, albeit in the context of prostate disease, our bacterial count results at six months do not appear unusual. In future experiments, shorter monthly follow ups to monitor changes in bacterial levels and associated pathological changes will be more informative.

Now that we have established a method for successful infection of sheep discs with *C. acnes*, our results will aid in the planning and design of future experiments with a greater number of animals. Further work should immediately focus on inoculum concentration, comparison of different strains, post-infection incubation periods, host inflammatory responses and analysis of IVD tissue for biofilm-like colonization and intracellular forms similar to that seen in other chronic diseases associated with *C. acnes* (e.g., sarcoidosis; prostate disease).

## 5. Conclusions

This proof-of-concept study demonstrates that sheep discs can be safely injected with *C. acnes* for in vivo study. Further work is now warranted with an increased number of animals and outcome measures to fully clarify the growth pattern and duration of *C. acnes* in ovine discs, and to further elucidate its role in the aetiology of disc degeneration. It is hoped that sheep will ultimately prove a good model to study the role of *C. acnes* in the pathophysiology of DDD, and to investigate the use of antibiotics and other novel therapeutic strategies to treat the condition in humans.

## Data Availability

Data is contained within the article and Supplementary Material.

## Supplementary Materials

The following are available online at https://www.mdpi.com/2306-7381/8/3/48/s1, Figure S1: Discograms verifying the accuracy of this percutaneous intradiscal lumbar spine injection technique. Figure S2: 25 gauge needles within the 20 gauge introducer needles, serially spaced, extending into the lumbar discs.
