# Supplementary material for: Sheep as a Potential Model of Intradiscal Infection by the Bacterium Cutibacterium acnes
Source: Vet Sci. 2021 Mar 16;8(3):48. doi: 10.3390/vetsci8030048 (PMC8002071; doi:10.3390/vetsci8030048)
Supplement: Supplementary file 1 [file vetsci-08-00048-s001.zip › vetsci-1148638-supplementary.pptx]

## Slide 1
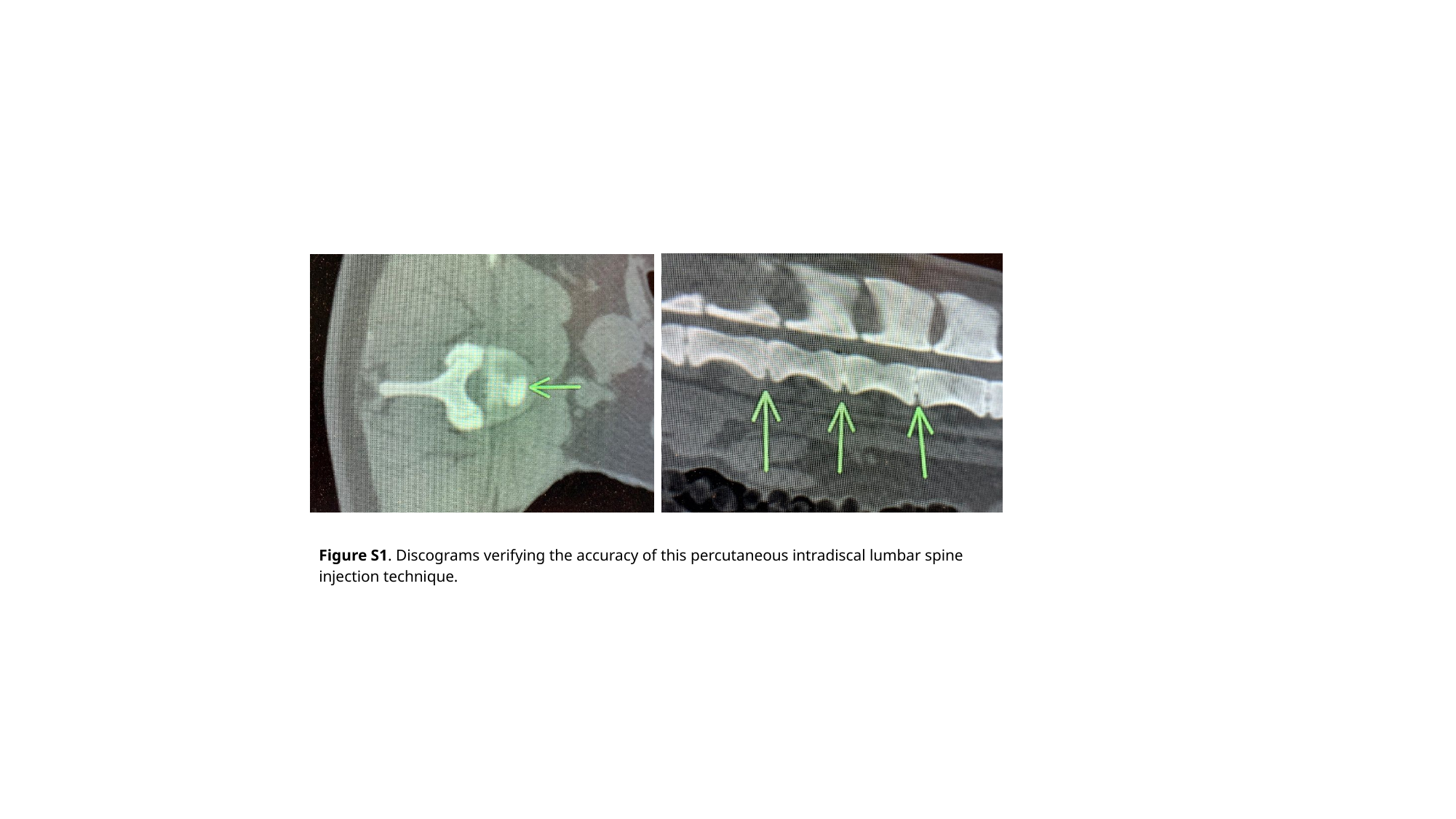

Figure S1. Discograms verifying the accuracy of this percutaneous intradiscal lumbar spine injection technique.

## Slide 2
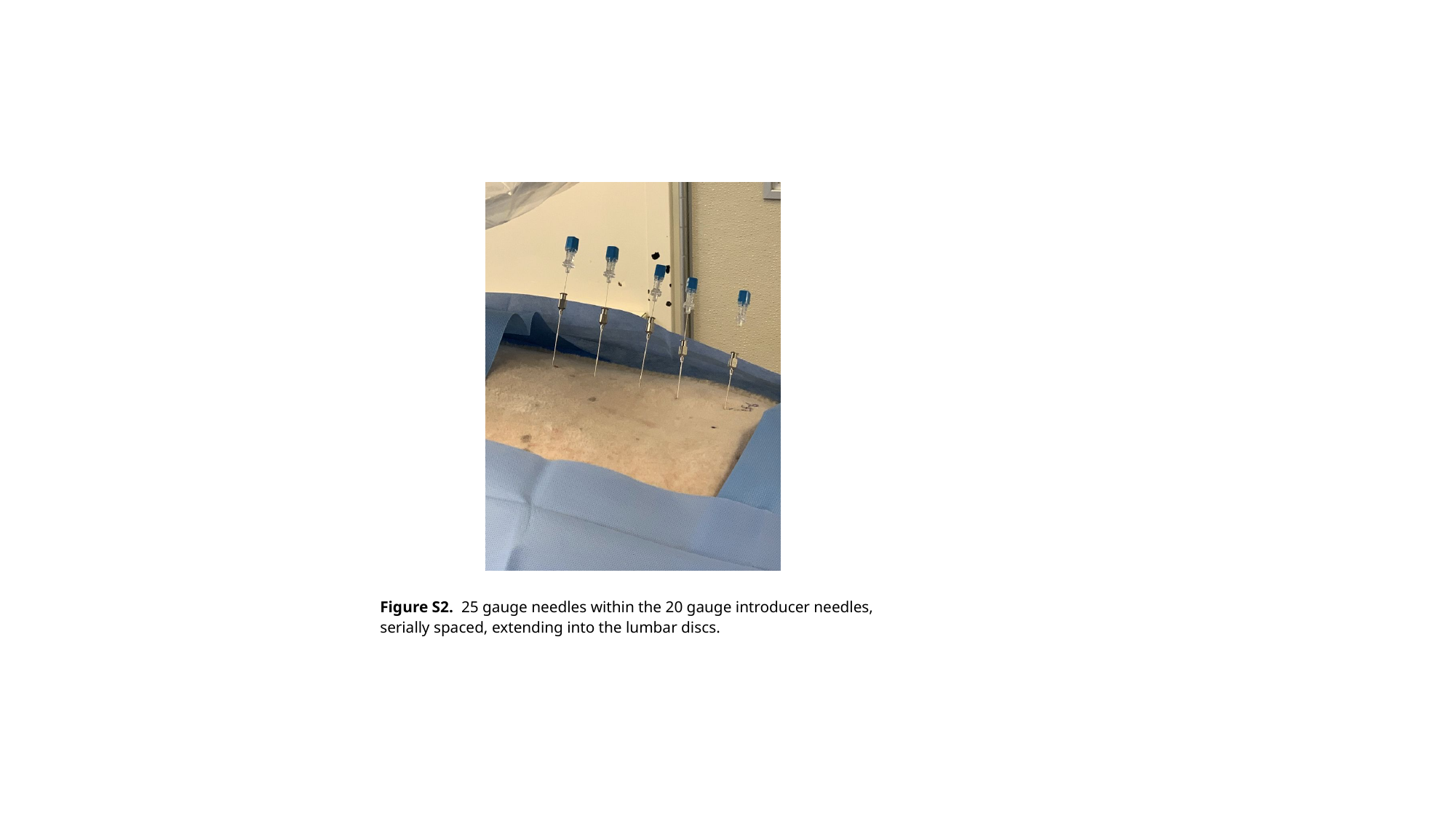

Figure S2. 25 gauge needles within the 20 gauge introducer needles, serially spaced, extending into the lumbar discs.
